# Glucometabolic Efficacy of the Empagliflozin/Metformin Combination in People with Type 1 Diabetes and Increased Cardiovascular Risk: A Sub-Analysis of a Pilot Randomized Controlled Trial

**DOI:** 10.3390/jcm13226860

**Published:** 2024-11-14

**Authors:** Miodrag Janić, Andrej Janež, Mišo Šabović, Mohamed El-Tanani, Imran Rangraze, Manfredi Rizzo, Mojca Lunder

**Affiliations:** 1 Department of Endocrinology, Diabetes and Metabolic Diseases, University Medical Centre Ljubljana, 1000 Ljubljana, Slovenia; andrej.janez@kclj.si (A.J.); mojca.lunder@kclj.si (M.L.); 2 Faculty of Medicine, University of Ljubljana, 1000 Ljubljana, Slovenia; miso.sabovic@kclj.si; 3 School of Medicine, Promise Department of Health Promotion Sciences Maternal and Infantile Care, Internal Medicine and Medical Specialties, University of Palermo, 90133 Palermo, Italy; manfredi.rizzo@unipa.it; 4 Department of Vascular Diseases, University Medical Centre Ljubljana, 1000 Ljubljana, Slovenia; 5 College of Pharmacy, Ras Al Khaimah Medical and Health Sciences University, Ras Al Khaimah P.O. Box 11172, United Arab Emirates; eltanani@rakmhsu.ac.ae; 6 Internal Medicine Department, Ras Al Khaimah College of Medical Sciences, Ras Al Khaimah Medical and Health Sciences University, Ras Al Khaimah P.O. Box 11172, United Arab Emirates; imranrashid@rakmhsu.ac.ae

**Keywords:** type 1 diabetes, empagliflozin/metformin combination, glycemic control, metabolic parameter improvement

## Abstract

**Background/Objectives**: People with type 1 diabetes have an unmet need for cardiovascular protection due to the lack of new recommended antidiabetic therapies with cardiovascular benefits. We examined whether the addition of an empagliflozin/metformin combination, and each drug alone, can complement insulin to improve glucometabolic parameters in overweight people with type 1 diabetes at high cardiovascular risk. **Methods**: This pilot, single-center double-blind randomized controlled trial included 40 people with type 1 diabetes. In addition to insulin, they received empagliflozin (25 mg daily), metformin (2000 mg daily), an empagliflozin/metformin combination, or a placebo. The intervention period was 12 weeks. Glycemic parameters, insulin requirements, and blood and urine samples were analyzed. Indices for liver fibrosis were calculated. Due to potential safety concerns, participants regularly measured blood ketone values. **Results**: The empagliflozin/metformin combination decreased HbA1c (−0.6%, *p* < 0.05) and weight (−6.1 kg, *p* < 0.05). Empagliflozin decreased the urinary albumin-to-creatinine ratio (−31.4 ± 4.9%, *p* = 0.002). The empagliflozin/metformin combination and empagliflozin decreased the estimated daily proteinuria (−34.6 ± 5.0%, *p* = 0.006 and −35.9 ± 6.2%, *p* = 0.03, respectively), the calculated FIB-4 (up to −17.8 ± 5.2%, *p* = 0.04 and −10.7 ± 3.7%, *p* = 0.02, respectively), and other liver fibrosis indices and uric acid values. No significant side effects occurred during the study. **Conclusions**: The empagliflozin/metformin combination improved glycemic control, reduced weight and insulin requirements, and produced several additional beneficial metabolic effects in overweight people with type 1 diabetes with increased cardiovascular risk.

## 1. Introduction

Individuals with type 1 diabetes have an increased risk of cardiovascular events, as shown by their on average 11–13-year shorter life expectancy compared to the general population [1]. Even with adequately managed glycemia, their risk of dying from cardiovascular causes is double that of people without diabetes [2]. The factors that increase cardiovascular risk in people with type 1 diabetes are similar to those found in type 2 diabetes (genetics, smoking, arterial hypertension, dyslipidemia, overweight/obesity, diabetic kidney disease, prolonged hyperglycemia exposure) and some specific to type 1 diabetes, such as a dysfunctional immune system [1,3]. Current recommendations to mitigate cardiovascular risk in people with type 1 diabetes include non-smoking, glycemic control, lipid reduction, arterial hypertension control, antithrombotic therapy, and treatment of overweight/obesity [1].

Since 2015, when the EMPA-REG OUTCOME trial was first published, data have accumulated showing that in people with and without type 2 diabetes and chronic kidney disease, treatment with sodium–glucose cotransporter 2 (SGLT-2) inhibitors results in reduced cardiovascular risk (HR: 0.76; [95% CI: 0.72–0.79]) and risk of a primary renal outcome (HR: 0.69; [95% CI: 0.61–0.79]) [4]. Furthermore, SGLT-2 inhibitors, regardless of diabetes status, reduce the composite outcomes of cardiovascular death or first hospitalization for heart failure (HR: 0.80 [95% CI: 0.73–0.87]), these results being consistent in both components [5]. It should be emphasized that in all these clinical trials, people with type 1 diabetes were excluded. In recent years, clinical trials have been conducted that added SGLT-2 inhibitors to insulin therapy in people with type 1 diabetes. The results show beneficial glycemic efficacy, as well as possible kidney and cardioprotective properties, but with an elevated risk of diabetic ketoacidosis. On the other hand, a recent meta-analysis of 16 randomized controlled trials in 7192 people with type 1 diabetes showed that the addition of SGLT-2 inhibitors reduced the glycated hemoglobin (HbA1c), fasting plasma glucose, mean amplitude of glucose excursions, and total insulin dosage, while not reducing the risk of hypoglycemia. The risk of diabetic ketoacidosis did not increase significantly in 1 month [6]. However, previous safety concerns in this population have led authorities in Europe and the United States to revoke licenses for the use of these drugs in type 1 diabetes [7].

There is an unmet need for cardiovascular prevention in people with type 1 diabetes, as they are deprived of the benefits of new antihyperglycemic drugs, such as SGLT-2 inhibitors, which have been proven in other populations. It may be time to rethink, verify the data, and once again reassess whether the benefits still outweigh the risks of possible treatment with SGLT-2 inhibitors in people with type 1 diabetes, as the meta-analysis by Nan et al. also suggests [6]. Our study group has already shown that empagliflozin and particularly an empagliflozin/metformin combination improved arterial function in people with type 1 diabetes in addition to the antioxidant and anti-inflammatory action [8,9]. Therefore, the objective of this analysis was to explore whether adjunct therapy with an empagliflozin/metformin combination, as well as both drugs separately, in overweight people with type 1 diabetes with increased cardiovascular risk, could complement insulin therapy and allow for the achievement of better glycemic goals and beneficial metabolic effects.

## 2. Materials and Methods

### 2.1. Study Design

This was a pilot, double-blind, randomized interventional trial in 40 people with type 1 diabetes carried out in the Outpatient Diabetes Clinic of the University Medical Center Ljubljana, Ljubljana, Slovenia.

**Figure 1:**
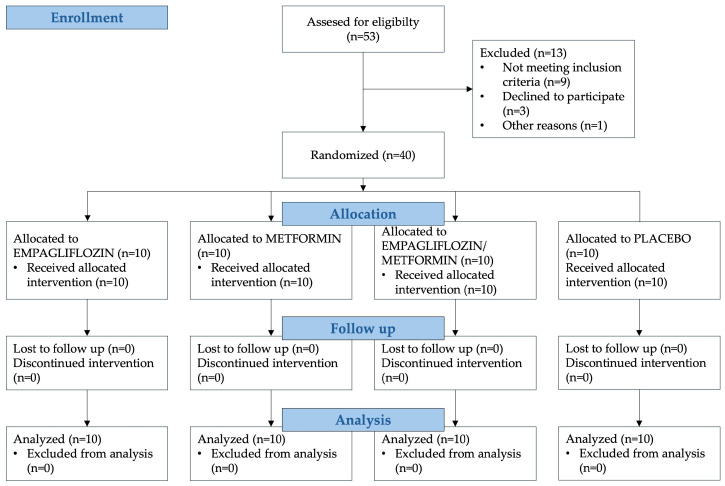
Flow diagram for study sample selection, allocation, follow-up, and analysis.

Of the 53 consecutive people with type 1 diabetes referred for potential inclusion into the study according to the previously distributed inclusion criteria, 3 did not meet the inclusion criteria, 9 declined to participate, and 1 was declined by the research team due to already questionable adherence to other therapy, thus allowing for equivalent intervention groups. CONSORT flow diagram is presented in Figure 1, please also see supplementary data [10].

The 40 participants were assigned to one of four treatment groups, based on a block randomization protocol. Each participant received a container with a number containing one of four interventional medications: (1) a placebo (control), (2) empagliflozin (25 mg daily), (3) metformin (2000 mg daily), and (4) empagliflozin/metformin (empagliflozin at 25 mg daily and metformin at 2000 mg daily). Interventional medications were administered in addition to insulin (multiple daily insulin injections or insulin pump therapy). The code of the contents of the containers was protected by a person not related to the study, so that the participants in the study and the investigators were blinded to the contents of the containers. The investigators received the code for inspection after the end of the data processing intervention period. None of the participants discontinued the intervention, so all data were analyzed. Adherence was not systematically followed.

All participants voluntarily participated in the study and signed informed consent prior to inclusion. The National Medical Ethics Committee of the Republic of Slovenia approved the study. This study is registered at “http://clinicaltrials.gov (accessed on 9 October 2024)” (NCT03639545).

### 2.2. Study Population

Inclusion criteria were a confirmed diagnosis of type 1 diabetes and an age between 30 and 65 years. Exclusion criteria comprised heart failure with a reduced ejection fraction (HFrEF), NYHA class II–III, NT-proBNP greater than 500 pg/mL, chronic kidney disease (estimated glomerular filtration rate (eGFR) < 60 mL/min/1.73 m^2^), advanced liver failure, benign prostatic hyperplasia, prostatic carcinoma, history of frequent urinary tract infections, or a body mass index (BMI) below 25 kg/m^2^.

### 2.3. Study Protocol

Participants were evaluated at the two time points during the study. At the time of inclusion in the study, a comprehensive medical history was taken, and a thorough medical examination was conducted. At inclusion and after 12 weeks, at the end of the study period, venous blood samples and second morning urine samples were collected for an additional analysis along with anthropometric assessments. Blood pressure was measured using an automated sphygmomanometer, before and after therapy.

### 2.4. Participant Safety Instructions

Participants were required to continue following their daily routine in the self-management of diabetes. Additionally, they were instructed to regularly measure blood ketone values daily after inclusion in the study. Blood ketone measurements were performed in the fasting state, before dinner, and in the case of increased blood glucose values, not feeling well, infection, or increased body temperature (Wellion Galileo Glu/Ket blood glucose and ketone monitoring system). Participants were ordered to immediately call researchers in case of positive blood ketones. They were also instructed not to aggressively decrease insulin doses or even omit insulin after being included in the study.

### 2.5. Anthropometric Measurements

All measurements were performed simultaneously by the same individual. Participants were uncovered, wearing only underwear. Each measurement was carried out twice, and if there was a notable difference between the two results, a third measurement was carried out. Body height was measured using the portable stadiometer, measuring the perpendicular distance between the top of the head and the bottom of the feet, the participants being barefoot. It was expressed in meters. Body weight was measured using a portable weighing scale and expressed in kilograms. The BMI was calculated as a division between body weight and squared body height. The waist circumference was assessed following the protocol, specifically as the girth of the abdomen in its thinnest section between the lower rib margin and the upper edge of the iliac crest, perpendicular to the long axis of the trunk, using an anthropometric tape measure.

### 2.6. Cardiovascular Risk Assessment

The average 5-year and 10-year risk of cardiovascular disease was calculated using the Steno risk engine for people with type 1 diabetes available at “https://steno.shinyapps.io/T1RiskEngine/ (accessed on 1 September 2018)”, based on [11]. According to the results of the 10-year cardiovascular disease risk, participants were classified as ‘high risk’, when the risk exceeded 20%; ‘medium risk’, when the risk was between 10 and 20%; or ‘low risk’, when the risk was less than 10%.

### 2.7. Continuous Glucose Monitoring Parameters and Daily Insulin Dose Determination

Continuous glucose monitoring (CGM) data were extracted from the different CGM devices in their users. Time in the range (TIR; percent of readings between 3.9 and 10 mmol/L), time below the range (TBR; percent of readings below 3.8 mmol/L), and time above the range (TAR; percent of readings above 10.1 mmol/L) were determined for the 12-week period. In participants who received multiple daily insulin injections, the total daily dose (TDD) of insulin was calculated by adding the prandial and basal doses. In those who received insulin pump therapy, pump data were extracted, and cumulative insulin doses were recorded.

### 2.8. Blood Sample and Urine Sample Analysis

Fasting venous blood samples were obtained at the beginning of the study and after 12 weeks of treatment. Blood glucose, HbA1c, and creatinine were determined using the VITRO 5.1FS Chemistry System (Ortho Clinical Diagnostics, Raritan, NJ, USA). The complete blood count was determined using an Advia 2010 analyzer (Siemens Healthcare, Erlangen, Germany). Cholesterol was measured using an enzymatic color test with chromophore detection at wavelength 505/649 nm. The HDL-cholesterol enzyme color test with cholesterol oxidase and cholesterol esterase with product detection at 596 nm was used. LDL cholesterol was analyzed through an enzyme color assay involving cholesterol oxidase and esterase. The hydrogen peroxide produced by these enzymes was measured using a Trinder endpoint reaction at 596 nm. Triglyceride levels were determined using a three-step enzymatic process with a Trinder endpoint product measured at 505/694 nm. Uric acid was assessed through an enzymatic reaction involving the detection of uricase, with the resulting product measured in a Trinder-like manner. The absorbance of the resulting complex was recorded at wavelengths of 545/694 nm. The alanine aminotransferase (ALT) and aspartate aminotransferase (AST) assays utilized pyridoxal-5′-phosphate, with a-ketoglutarate added to start the reaction. NADH concentrations were evaluated by measuring absorbance at 340/410 nm, where the decrease in absorbance rate was correlated with the activity levels of ALT or AST, respectively. Gamma-glutamyl transferase (γ-GT) was measured using a synthetic substrate (L-g-glutamyl-3-carboxy-4-nitroanilide). The formation rate of 5-amino-2-nitrobenzoate from the substrate was measured photometrically at 410/478 nm as a zero-order kinetic assay. For all analyses, the Advia 1800 Clinical Chemistry System from Siemens Healthcare Diagnostics, Siemens Healthineers, Erlagen, Germany was used. The quantitative determination of albumin in urine was based on the immunonephelometric method with specific antibodies in the Atellica NEPH 630 system (Siemens Healthcare Diagnostics Inc.). For the determination of urine proteins, the modified pyrogallol red-molybdate method was used. Urine creatinine was measured using the modified kinetic Jaffe reaction (both Dimension clinical chemistry systems; Siemens Healthcare Diagnostics). The calculation of the estimated glomerular filtration rate (eGFR) was performed utilizing the CKD-EPI equation.

### 2.9. Calculation of Liver Fibrosis Indices

The fibrosis-4 index for liver fibrosis (FIB-4), fatty liver index (FLI), and non-alcoholic fatty liver disease (NAFLD) fibrosis score were calculated for each participant using an automated calculator, based on previous relevant equations [12,13,14].

### 2.10. Statistical Analysis

Data were presented as the mean ± SEM. Variations were evaluated using a one-way analysis of variance (ANOVA). If interactions were significant, the Bonferroni post-test was conducted. A *p*-value for multiple comparisons was calculated, and less than 0.05 was deemed statistically significant. The statistical analysis was performed with GraphPad Prism version 7.0 software.

## 3. Results

### 3.1. Characteristics of the Participants

Participants were overall 44.7 ± 2.5 years old; the mean duration of type 1 diabetes was 22.6 ± 3.9 years. At inclusion, their average HbA1c was 7.8 ± 0.3%. The described parameters did not differ between the study groups at inclusion, as shown in Table 1. The empagliflozin/metformin combination significantly decreased body weight after 12 weeks of treatment (87.9 ± 1.5 kg vs. 81.8 ± 1.4 kg; *p* < 0.05), whereas BMI did not reach statistically significant differences (28.9 ± 0.9 vs. 27.7 ± 1.0 kg/m^2^; NS). The waist circumference and blood pressure values did not change significantly during the study period.

### 3.2. Calculation of the Cardiovascular Risk

The average 5-year cardiovascular risk was 15.1 ± 1.7%, while the average 10-year cardiovascular risk was 26.7 ± 2.6%. According to the results of the 10-year cardiovascular risk, 65% of the participants were classified as ‘high risk’, 25% as ‘medium risk’, and only 10% as ‘low risk’.

### 3.3. Glycaemic Efficacy and Daily Insulin Doses

**Figure 2:**
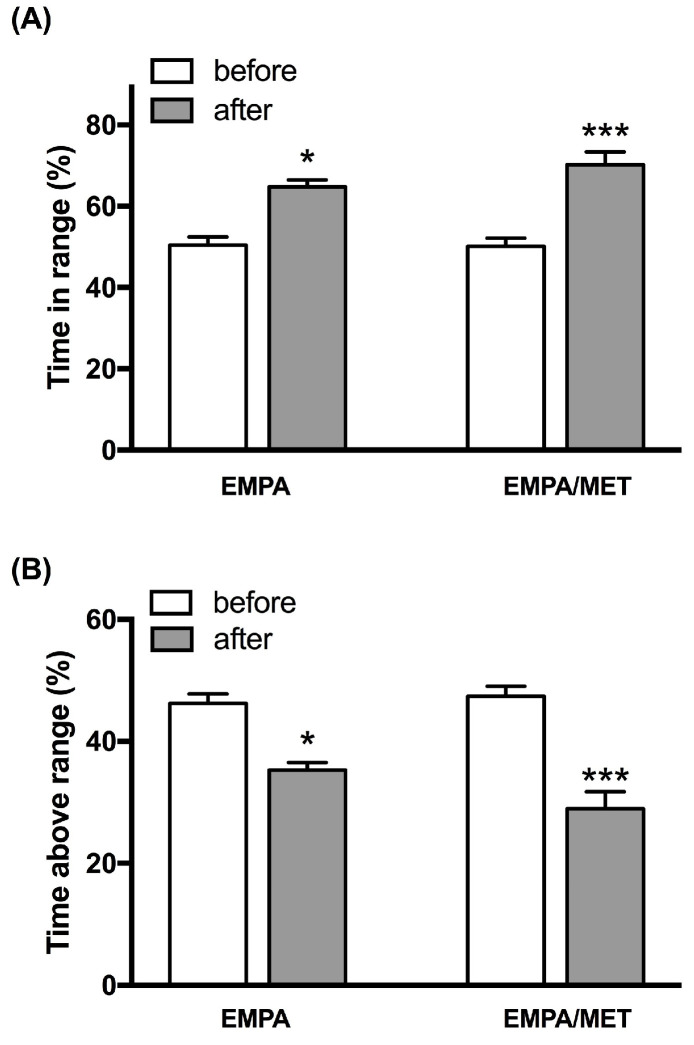
Changes in (**A**) time in range (TIR) and (**B**) time above range (TAR) in groups treated with empagliflozin (25 mg daily) (EMPA) or the empagliflozin/metformin combination (25 mg/2000 mg daily) (EMPA/MET). * *p* < 0.05 adjusted for multiple comparisons versus initial values; *** *p* < 0.001 adjusted for multiple comparisons versus initial values.

Only the empagliflozin/metformin combination significantly decreased HbA1c values (7.8 ± 0.2% vs. 7.2 ± 0.2%, *p* < 0.05), whereas both drugs separately did not significantly influence it. The addition of the empagliflozin/metformin combination or empagliflozin alone to standard insulin treatment significantly increased TIR (up to 15% (*p* < 0.05) or 20% (*p* < 0.001), respectively; Figure 2) and decreased TAR (up to −11% (*p* < 0.05) or −18% (*p* < 0.001), respectively; Figure 2), compared to initial values. The TBR remained unchanged during the course of the study.

The addition of metformin, empagliflozin, or the empagliflozin/metformin combination reduced the prandial and basal daily insulin doses, as well as insulin TDD after 12 weeks of treatment. In participants receiving the empagliflozin/metformin combination, insulin doses decreased significantly compared to controls (prandial daily insulin dose up to −22.2 ± 1.2%, *p* < 0.001; basal daily insulin dose up to −7.4 ± 0.9%, *p* = 0.0038; and TDD up to −28.4 ± 0.9%, *p* < 0.001). Separate drugs were less effective. No changes in prandial or basal insulin doses were observed in the control group.

### 3.4. Renal and Metabolic Laboratory Parameters

#### 3.4.1. Renal Function and Uric Acid Levels

During treatment with an empagliflozin/metformin combination or separate drugs, renal function was preserved. Serum creatinine values and eGFR did not change during the course of the study (Table 2). The empagliflozin and the empagliflozin/metformin combination significantly decreased estimated daily proteinuria (eDP) (up to −35.9 ± 6.2%, *p* = 0.03 and −34.6 ± 5.0%, *p =* 0.006, respectively), while metformin alone did not affect it. Empagliflozin also significantly decreased the urinary albumin-to-creatinine ratio (UACR; up to −31.4 ± 4.9%, *p =* 0.002) (Table 2). The combination of empagliflozin/metformin or empagliflozin alone significantly decreased uric acid values (up to −12.9 ± 2.0%, *p* < 0.05 and −13.1 ± 2.9%, *p <* 0.05, respectively), while metformin alone had no influence (Table 2).

#### 3.4.2. Liver Function Tests

The empagliflozin/metformin combination or separate drugs did not influence AST, ALT, and γ-GT values (Table 3). The empagliflozin/metformin combination and empagliflozin alone significantly decreased the calculated FIB-4 (up to −17.8 ± 5.2%, *p* = 0.04 and −10.7 ± 3.7%, *p* = 0.02, respectively). Similarly, the empagliflozin/metformin combination and empagliflozin alone significantly decreased the calculated FLI (up to −36.7 ± 9.8%, *p* = 0.003 and −25.4 ± 7.1%, *p* = 0.002, respectively). Furthermore, the combination of empagliflozin/metformin and empagliflozin alone also decreased the score of NAFLD fibrosis (up to −43.7 ± 11.2%, *p* < 0.001 and −31.2 ± 9.3%, *p* = 0.02). In contrast, metformin alone did not affect the FIB-4, FLI, or NAFLD fibrosis scores (Table 3).

#### 3.4.3. Blood Lipid Values

The empagliflozin/metformin combination or separate drugs did not influence the absolute values of blood lipids. When comparing relative changes during 12 weeks of treatment, metformin significantly decreased total cholesterol (up to −10.8 ± 3.1%, *p* < 0.01, versus control) and LDL (up to −12.3 ± 4.1%, *p* = 0.0029, versus control), while HDL values and triglycerides remained unchanged. Empagliflozin increased LDL cholesterol values (up to +7.6 ± 2.1%, NS), this effect being nullified by adding metformin. In the control group, no changes in lipid values were observed during the study.

### 3.5. Safety Concerns

No increase in the number of urogenital infections was observed. There were no episodes of diabetic ketoacidosis during the study course; participants regularly measured ketone levels in their blood and the values remained low throughout the study. Furthermore, no increase in the incidence of hypoglycemia (mild or severe) was reported during the study.

## 4. Discussion

In the present pilot study, our aim was to explore whether adjunct therapy with an empagliflozin/metformin combination or separate drugs could beneficially complement insulin therapy and allow greater glycemic control and beneficial metabolic effects in overweight people with type 1 diabetes with increased cardiovascular risk. Treatment with the empagliflozin/metformin combination significantly improved glycemic parameters after 12 weeks of treatment, that is, a reduction in HbA1c, while separate drugs did not [8]. The empagliflozin/metformin combination as well as empagliflozin alone also increased TIR, while concomitantly reducing TAR. TBR did not change significantly in any treatment group. Additionally, a significant weight reduction was observed in the combination treatment group, but not in separate groups. These observations were accompanied by a significantly reduced prandial dose, basal dose, and TDD of insulin in all three treatment groups, but not at the expense of safety considerations, as no diabetic ketoacidosis or severe hypoglycemia were detected. Interestingly, renal protection was recorded in terms of reduction in eDP in both groups treated with empagliflozin or the empagliflozin/metformin combination, while reduction in UACR was detected only in the empagliflozin-treated group. Furthermore, uric acid levels decreased significantly in the empagliflozin- and empagliflozin/metformin-treated groups, while metformin alone did not show an effect. Liver protection, expressed as improvements in the FIB-4, FLI, and NAFLD fibrosis score, was also significantly recorded in the empagliflozin group and the group treated with the empagliflozin/metformin combination. Improvements in three scoring systems show consistency in liver protection effects led by empagliflozin. These results provide information on the metabolic effects of adjunct oral therapy in overweight people with type 1 diabetes, especially by shedding light on the favorable effects of the empagliflozin/metformin combination. Therefore, we believe that the addition of the empagliflozin/metformin combination to insulin treatment could be beneficial in the group of people with type 1 diabetes characterized by increased weight, elevated cardiovascular risk, and poorly controlled glycemia. This therapeutic regime appears to be safe and well tolerated in our study cohort.

This study was designed as a sub-analysis of our previous studies that showed beneficial protective effects of the empagliflozin/metformin combination on several parameters of arterial function in people with type 1 diabetes, which have predictive values for future cardiovascular events [8]. The combination was also shown to have significant antioxidant and anti-inflammatory effects that were correlated with improved arterial function [9]. However, from a clinician’s point of view, the current sub-analysis could show that the empagliflozin/metformin combination induces beneficial glucometabolic effects in overweight people with type 1 diabetes and increased cardiovascular risk.

The idea of adjunctive oral antidiabetic therapy with insulin therapy in people with type 1 diabetes is not new. Several drugs have been tested in this setting with the aim of reducing HbA1c and improving/reducing glycemic variability. Furthermore, other potential beneficial effects of adjuvant drugs could have been expected. In the EASE (Empagliflozin as Adjunctive to inSulin thErapy) trials, empagliflozin at 10 and 25 mg, as well as a unique dose of 2.5 mg, in addition to intensive insulin therapy in people with type 1 diabetes was evaluated. The 26-week study showed in 1707 people with type 1 diabetes that empagliflozin improved glycemic control and decreased weight, without increasing the risk of hypoglycemia. The effect on HbA1c was −0.53% (−0.65, −0.42) for 25 mg [15], similar to our study for the empagliflozin/metformin combination, while the single drug did not achieve a similar improvement in glycemic control. However, we observed a greater reduction in insulin TDD than in the EASE trials for empagliflozin alone, which was similar to the effect of metformin, while the reduction in insulin TDD for combination therapy reached a significant −28.4 ± 0.9%. EASE trials also showed an incidence in diabetic ketoacidosis of 3.3% [15], while we do not report ketoacidosis. Lu and colleagues performed a meta-analysis of 10 studies, concluding that the combination of SGLT-2 inhibitors with insulin treatment was beneficial in people with type 1 diabetes. They concluded that SGLT-2 inhibitors decreased HbA1c and reduced body weight [16], which was observed in our study only in the group treated with the empagliflozin/metformin combination. These differences for separate drugs could be attributed to the shorter duration of the study and the much smaller study population in our study. But, on the other hand, our study shows that the effect with the empagliflozin/metformin combination could be achieved even after 3 months. Furthermore, SGLT-2 inhibitors reduced daily insulin requirements, which is consistent with our results [15]. Similar results were also shown in a meta-analysis of several studies exploring the effects of different SGLT-2 inhibitors in people with type 1 diabetes [17]. A recent meta-analysis by Nan et al. reports that the addition of SGLT-2 inhibitors to insulin therapy in 7192 people with type 1 diabetes significantly but gradually reduced HbA1c, while reduction in body weight was the biggest in the 2–6-month period, after a plateau was reached [6]. Our results agree with these findings for the combination treatment, but not for empagliflozin alone, and we cannot claim whether this would be the greatest reduction in the parameters mentioned. On the other hand, like the results of Nan et al. [6], we also observed a significant reduction in insulin doses in all treated groups without an increased risk of diabetic ketoacidosis or hypoglycemia. Although a recent meta-analysis reports an increased risk of diabetic ketoacidosis with an increase in the duration of treatment [6], we believe that strict patient education minimizes this risk. In our study, urinary tract and genital infections were not reported. Furthermore, metformin has also been shown to reduce insulin doses in people with type 1 diabetes [18,19], which is consistent with the results of our study, but this finding was not uniformly observed in all studies [20]. In several studies, metformin also decreased body weight [19,20]. Studies were performed comparing the effect of adjunctive use of SGLT-2 inhibitors and metformin in people with type 1 diabetes, the comparison being indirect [21] or direct [22], showing a consistent improvement in glycemic control and a reduction in body weight, blood pressure, and insulin TDD with the former compared to metformin [21,22]. However, to date, we are unaware of a study showing the effect of the empagliflozin/metformin combination on the parameters mentioned above.

Several previous studies described the renal protective effects of SGLT-2 inhibitors. Most studies showing the renoprotective effects of SGLT-2 inhibitors included people with type 2 diabetes [23,24], while a recent meta-analysis by Karakasis et al. showed that using SGLT-2 inhibitors as an additional treatment in individuals with type 1 diabetes led to a notable decrease in albuminuria, whereas their usage had a neutral impact on creatinine clearance [25]. For metformin and renal protection, the results of the studies are divergent; renoprotective effects were observed in some studies [26,27] but not in others [28]. These results are in agreement with our study for empagliflozin alone, although the empagliflozin/metformin combination did not show a significant effect on the reduction in UACR, but there was an evident trend towards its reduction.

Even people with type 1 diabetes could have dysmetabolic derangements, such as overweight/obesity, metabolic dysfunction-associated steatotic liver disease (MASLD), atherogenic dyslipidemia, hyperuricemia, etc. A recent meta-analysis in people with type 2 diabetes showed that there could be a slight improvement in liver steatosis and/or fibrosis compared to controls based on imaging and histopathological biomarkers, but with a low-to-moderate certainty of evidence [29,30]. However, there is no study showing this effect in people with type 1 diabetes, but a study showed protective effects on the liver in rats with type 1 diabetes [31]. Metformin also showed protective effects in MASLD, but the results of studies were again contradictory [32]. In this study, we observed a favorable effect in the group of participants treated with empagliflozin as well as the empagliflozin/metformin combination, as shown with consistency in three scoring indices of liver pathology.

This study evaluated the adjunctive therapy of the empagliflozin/metformin combination in overweight people with type 1 diabetes and increased cardiovascular risk. The results found agree with previous findings for the separate drugs, while the combination could lead to superior results. As shown, especially the empagliflozin/metformin combination improved glycemia control and reduced body weight, while also improving important metabolic parameters in people with type 1 diabetes, all of which indicate increased cardiovascular risk. Therefore, these findings refer to the fact that both drugs in combination could be more beneficial in reducing cardiovascular risk in people with type 1 diabetes, compared to each drug separately. Furthermore, the results that prioritize the empagliflozin/metformin combination are further strengthened by our previous finding that the empagliflozin/metformin combination significantly improved arterial functions and reduced oxidative stress and inflammation in people with type 1 diabetes [8,9]. Therefore, the proposal to add the empagliflozin/metformin combination for use in overweight people with type 1 diabetes with increased cardiovascular risk appears to be logical and based on clear benefits beyond better glycemic control.

It is well known that adherence to therapy is a particular problem in people with diabetes. In light of this problem, it is important to consider the well-known clinical fact that people who reported better glycemia control also reported better self-satisfaction, which in turn provided positive feedback in terms of adherence [32]. This could be an additional benefit of the empagliflozin/metformin combination. Furthermore, it is of particular importance that participants in the present study reported no more hypoglycemia events than previously anticipated, and no diabetic ketoacidosis events were reported. However, this pilot study has several limitations. The first is the small number of participants, resulting in relatively high variability in observed parameters, and therefore, not showing significance everywhere expected. Second, because of the small number of participants, these results have limited generalizability. Third, this could also lead to positive or false-negative results. Fourth, eventual errors could also have been attributed to potential non-adherence. Fifth, there could be selection bias, as study participants were randomly selected and assigned to a specific treatment group. Sixth, a significant proportion of participants in the metformin and control groups did not use a sensor system, thus preventing a reliable statistical analysis of the sensor parameters.

Undoubtedly, well-designed and adequately powered randomized controlled trials with the addition of an empagliflozin/metformin combination to insulin therapy are warranted in people with type 1 diabetes and a cardiovascular outcome trial that demonstrates these observations with respect to the reduction in major cardiovascular events.

## 5. Conclusions

In this exploratory pilot study, we found that the empagliflozin/metformin combination could have several potential glucometabolic benefits in overweight people with type 1 diabetes and increased cardiovascular risk. These potential benefits are reflected in an improvement in glycemic control and metabolic parameters that culminates in a reduction in HbA1c, body weight, and insulin doses, as well as the probable protection of the kidney and liver. Although these potential benefits complement the effects of the empagliflozin/metformin combination previously shown on arterial function, they need to be investigated in larger well-designed and adequately powered studies.

## Figures and Tables

**Table 1:** Baseline clinical characteristics of the participants at the time of study enrollment, stratified by study groups.

	CTRL (*n* = 10)	EMPA (*n* = 10)	MET (*n* = 10)	EMPA/MET (*n* = 10)
Average age (years)	43.1 ± 2.1	46.0 ± 2.3	46.4 ± 3.9	43.3 ± 2.6
Duration of type 1 diabetes (years)	22.2 ± 3.8	22.5 ± 3.7	23.1 ± 4.8	22.3 ± 3.2
Body weight (kg)	88.1 ± 2.0	87.0 ± 3.1	86.9 ± 1.8	87.9 ± 1.5
BMI (kg/m^2^)	28.3 ± 0.5	28.9 ± 0.7	28.0 ± 0.3	28.9 ± 0.9
Waist circumference (cm)	102.5 ± 2.6	104.2 ± 2.1	101.8 ± 3.3	105.1 ± 3.5
HbA1c (%)	7.8 ± 0.2	7.8 ± 0.1	7.9 ± 0.2	7.8 ± 0.2
Systolic blood pressure (mmHg)	129.9 ± 4.0	134.5 ± 3.0	132.4 ± 3.1	129.9 ± 2.6
Diastolic blood pressure (mmHg)	73.2 ± 5.4	83.8 ± 2.6	79.1 ± 1.5	84.4 ± 1.1
Heart rate (beats/min)	81.7 ± 1.7	80.2 ± 1.9	79.8 ± 2.0	80.3 ± 1.6
Smoking (*n*)	2	2	2	2
Arterial hypertension (*n*)	4	3	3	4
Dyslipidemia (*n*)	2	3	2	3
Diabetic retinopathy (*n*)	4	5	4	4
Diabetic neuropathy (*n*)	1	2	1	1

Data are presented as mean values accompanied by the standard error of the mean. The absolute number (*n*) of participants experiencing each condition (smoking, arterial hypertension, dyslipidemia, diabetic retinopathy, diabetic neuropathy) is specified. CTRL—control group; EMPA—empagliflozin group; MET—metformin group; EMPA/MET—empagliflozin/metformin group; BMI—body mass index; HbA1c—glycated hemoglobin.

**Table 2:** Renal parameters and uric acid levels.

	CTRL Before	CTRL After	EMPA Before	EMPA After	MET Before	MET After	EMPA/MET Before	EMPA/MET After
Creatinine (μmol/L)	84.9 ± 4.9	87.1 ± 5.5	86.2 ± 2.4	85.2 ± 2.2	86.3 ± 4.1	84.9 ± 4.7	85.1 ± 1.3	86.6 ± 2.2
eGFR (mL/min/1.73 m^2^)	83.6 ± 2.4	82.9 ± 2.7	85.7 ± 1.9	86.3 ± 1.9	81.6 ± 2.8	80.7 ± 2.8	87.9 ± 2.0	85.1 ± 1.4
eDP (mg/day)	0.27 ± 0.06	0.26 ± 0.05	0.27 ± 0.04	0.16 ± 0.02 *	0.30 ± 0.07	0.30 ± 0.08	0.33 ± 0.08	0.20 ± 0.04 **
UACR (g/mol)	5.23 ± 1.0	5.3 ± 1.1	4.9 ± 1.6	1.6 ± 0.3 *	5.5 ± 0.9	5.1 ± 0.9	6.0 ± 1.1	4.8 ± 0.8
Uric acid (µmol/L)	283.5 ± 9.7	273.5 ± 8.2	292.6 ± 10.5	264.2 ± 10.8 *	289.8 ± 13.9	291.3 ± 10.7	294.0 ± 7.5	263.9 ± 8.1 *

CTRL—control group; EMPA—empagliflozin group; MET—metformin group; EMPA/MET—empagliflozin/metformin group; eGFR—estimated glomerular filtration rate; eDP—estimated daily proteinuria; UACR—urinary albumin-to-creatinine ratio. * *p* < 0.05 and ** *p* < 0.01 adjusted for multiple comparisons before and after treatment in separate groups.

**Table 3:** Liver function parameters and the FIB-4 index.

	CTRL Before	CTRL After	EMPA Before	EMPA After	MET Before	MET After	EMPA/MET Before	EMPA/MET After
AST (µkat/L)	0.37 ± 0.01	0.36 ± 0.02	0.39 ± 0.02	0.40 ± 0.02	0.42 ± 0.02	0.39 ± 0.02	0.43 ± 0.04	0.38 ± 0.03
ALT (µkat/L)	0.50 ± 0.02	0.49 ± 0.02	0.52 ± 0.02	0.50 ± 0.02	0.52 ± 0.02	0.48 ± 0.03	0.50 ± 0.02	0.50 ± 0.03
γ-GT (µkat/L)	0.33 ± 0.03	0.31 ± 0.03	0.26 ± 0.01	0.26 ± 0.02	0.32 ± 0.03	0.33 ± 0.03	0.29 ± 0.02	0.27 ± 0.02
FIB-4	0.84 ± 0.03	0.80 ± 0.03	0.84 ± 0.03	0.75 ± 0.04 *	0.89 ± 0.05	0.88 ± 0.05	0.90 ± 0.10	0.74 ± 0.05 *
FLI	1.54 ± 0.13	1.51 ± 0.12	1.62 ± 0.08	1.15 ± 0.10 **	1.51 ± 0.08	1.50 ± 0.08	1.58 ± 0.10	0.91 ± 0.06 **
NAFLD fibrosis score	−0.8 ± 0.2	−0.9 ± 0.1	−0.9 ± 0.2	−1.3 ± 0.1 *	−0.8 ± 0.2	−0.9 ± 0.2	−0.8 ± 0.3	−1.2 ± 0.2 ***

CTRL—control group; EMPA—empagliflozin group; MET—metformin group; EMPA/MET—empagliflozin/metformin group; AST—aspartate aminotransferase; ALT—alanine aminotransferase; γ-GT—gamma-glutamyl transferase; FIB-4—fibrosis-4 index for liver fibrosis; FLI—fatty liver index; NAFLD—non-alcoholic fatty liver disease. * *p* < 0.05; ** *p* < 0.01; and *** *p* < 0.001 adjusted for multiple comparisons before and after treatment in separate groups.

## Data Availability

All data relevant to the study are included in the article. Data sets analyzed in this study can be shared, upon reasonable request, from the journal. However, it can be limited due to patient privacy and ethical restrictions.

## Supplementary Materials

The following supporting information can be downloaded at: https://www.mdpi.com/article/10.3390/jcm13226860/s1, The CONSORT checklist is appended in the supplementary material.
